# Association between downhill mountain bike racing and neurophysiological indices of sensory, attentional and cognitive processing in youth athletes

**DOI:** 10.1016/j.jsampl.2026.100152

**Published:** 2026-07-21

**Authors:** Matthew G. Neill, Elizabeth KS. Fletcher, Ember Larson, Carolyn A. Emery, Jonathan D. Smirl

**Affiliations:** a Sport Injury Prevention Research Centre, Faculty of Kinesiology, University of Calgary, Calgary, Alberta, Canada; b Cerebrovascular Concussion Laboratory, Faculty of Kinesiology, University of Calgary, Calgary, Alberta, Canada; c Alberta Children's Hospital Research Institute, University of Calgary, Calgary, Alberta, Canada; d Hotchkiss Brain Institute, University of Calgary, Calgary, Alberta, Canada; e Human Performance Laboratory, Faculty of Kinesiology, University of Calgary, Calgary, Alberta, Canada; f Libin Cardiovascular Institute of Alberta, University of Calgary, Alberta, Canada

**Keywords:** Neurophysiology, Event related potentials, Mountain biking, Youth

## Abstract

**Background:**

There is growing concern over potential neurological impairment associated with accumulation of repetitive head acceleration events (HAE) in sport, particularly during periods of neurological vulnerability, including youth. Despite the high volume of HAEs sustained during downhill mountain biking, it is currently unknown downhill mountain biking is associated with neural function in youth. Thus, the purpose of this study was to investigate the association between exposure to downhill mountain bike racing and sensory, attentional, and cognitive processing in youth using N100, P300 and N400 Event related Potentials (ERPs).

**Methods:**

Fifteen participants (6 female, 9 male; ages 15–24 years) contributed 38 ERP observations at baseline timepoints and following up to 5 days of elite downhill mountain bike competition.

**Results:**

Conditional linear regression models demonstrated that the average marginal difference in N400 amplitude in measures taken post-mountain bike exposure was 1.26 μV (95% CI: −2.16, −0.36; p = 0.006) less than before riding. N400 latency, P300 amplitude and latency, and N100 amplitude and latency were unchanged from baseline by days (range: 1–4) of mountain biking (all p > 0.26).

**Conclusion:**

In agreement with studies in hockey and football, our results showed that participation in downhill mountain biking is associated with acute cognitive neural processing impairments. The contribution of exercise, focus and HAEs to these impairments is unclear. This study expands the growing evidence of neural processing impairments occurring acutely following sport participation to the understudied sport of downhill mountain biking.

**Key Points:**

• Mountain biking is a popular sport that elicits an exercise response, repetitive head acceleration events, and requires focus.
• We demonstrated that cognitive neural processing (N400 amplitude) was reduced following downhill mountain biking
• Sport participation is associated with neurological changes even when acute brain injury does not occur, though the relative contribution of exercise, repetitive head impact and mental activity exposures is unclear.

## 1 Introduction

Sport participation in youth is associated with improvements in physical and mental health outcomes [1], though it is also the primary setting for youth injury [2]. The sporting landscape is evolving, and a host of new individual outdoor sports are increasing in popularity. Sporting contexts are promising for the development of adolescents and youth but also introduces new challenges in injury prevention, associated with informal or developing governing bodies, new equipment, and incompletely understood sporting demands.

Novel sports such as downhill mountain biking (MTB) are increasingly popular sports in North American contexts, with 17.2% of Canadians between the ages of 15 and 24 years of age reporting some participation in the last 12 months [3]. The widespread emergence of sports such as MTB have left an evidence gap in current sport-related health research. MTB has been demonstrated to elicit repetitive head acceleration events (HAEs) [4]. Moreover, there is growing concern that cumulative exposure to repetitive HAEs may be associated with short-term neurological dysfunction [5] and long-term neurodegeneration [6]. Exposure to repetitive HAEs is associated with an increased hazard of death [7] and predisposes athletes to concussion [8,9]. Exposure to HAEs during sport has been associated with impaired cerebrovascular function [10], white matter activity, neuroinflammation [11] and neurophysiological function [12,13]. Moreover, a recent study demonstrated acute bouts of MTB were associated with reductions in executive processing compared to laboratory-based exercise [14]. However, studies directly assessing measures of neurological function following MTB in a field-based setting are lacking.

Neurological function can be investigated by assessing event-related potentials (ERP) which are measures of the neural response to a standardised time-locked stimulus, recorded using electroencephalography (EEG). The amplitude of an ERP represents neurological resource allocation in response to the stimulus, while the latency indicates the speed at which the response was generated. ERPs are identified by their timing and the polarity of their response, with N100 and N400 ERPs representing negative deviations in neuroelectric wave forms around 100 and 400 ms, respectively, and the P300 being a positive neuroelectric deviation around 300 ms. ERPs can quantify neurophysiological indices of sensory processing with the N100 [15], basic attention with the P300 [16], and cognitive processing with the N400 responses [17,18]. Moreover, there is evidence of the N100, P300 and N400 ERPs being sensitive to neural activation changes associated with acute concussion [19], history of concussion [20] and contact/collision sport exposure [5,21,22]. There is evidence that N100 amplitudes were reduced and N400 latencies were extended in adolescents over the course of a season of contact sport exposure (e.g. rugby, ice hockey), relative to non-contact sport (e.g. rowing, tennis) participation [22] indicating underlying neurophysiological dysfunction potentially related to contact sport exposure.

Given the increasing popularity of mountain biking, it is of considerable interest to evaluate the association between MTB participation and potential neurological dysfunction. Thus, the purpose of this study was to investigate the association between exposure to competition days of downhill mountain bike racing and objective measures of sensory, attentional, and cognitive neural processing in elite youth athletes, assessed by the amplitude and latency of N100, P300 and N400 ERPs, respectively. We hypothesized that MTB exposure would be associated with acute neural dysfunction, manifesting as either a decrease in ERP amplitude or increase in ERP latency.

## 2 Methods

This prospective cohort study included a convenience sample of youth participants (aged 15–24) from an elite MTB competition event (Crankworx, Whistler, Canada). Data was collected in alignment with the longitudinal ‘Surveillance in Highschool and community sport to REDuce concussions and their consequences’ (SHRED Concussions) cohort study [23]. All participants provided informed consent prior to participation. The study underwent ethics review by the University of Calgary Conjoint Health Research Ethics Board (CHREB ID: REB21-0968, REB20-1662).

**Fig. 1:**
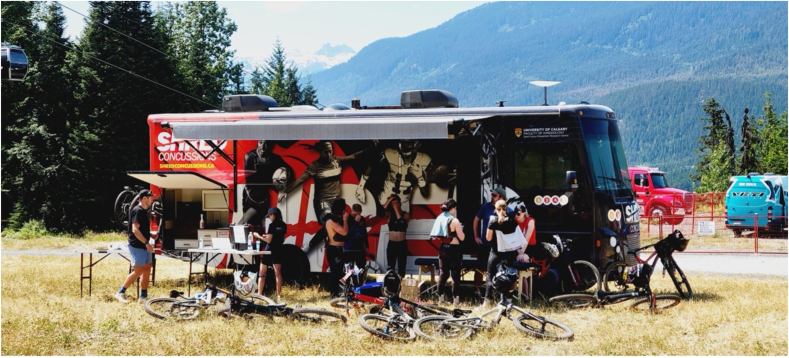
On-site data collection in the mobile ‘SHRedmobile’ laboratory.

Demographic information was collected directly in an online portal linked to a database via self-report (REDcap v.16.1.4) [24]. The cohort was tracked prospectively throughout the 2024 Crankworx downhill mountain bike competition. Neurological measures were collected on site in Whistler, AB using a mobile laboratory (the SHRedmobile) situated inside a class A Recreational Vehicle (RV) that was driven to the competition location (https://www.ucalgary.ca/sport-injury-prevention-research-centre/shred-mobile). Participants completed the neurological assessment at baseline and post-ride timepoints. Post-ride timepoints consisted of any timepoint after a baseline assessment, and whereby the athlete competed in a day of MTB on the day(s) of assessment. Total days of riding between baseline assessment and each subsequent assessment were recorded. Post-ride assessments were collected directly at the competition venue (Fig. 1) or in a building nearby the competition course, as available.

### 2.1 Neurological assessment

ERP outcomes were collected using a portable, point-of-care EEG system (NeuroCatch, Surrey, BC, Canada) that comprises 3 electrodes at midline locations (Fz, Pz, Cz), an electrooculogram (Fpz), a ground electrode (Afz) and a reference electrode on the right earlobe [25]. Prior to assessment, skin on the forehead and ear was cleaned using isopropyl alcohol wipes (LernaPharm, Montreal, QC, Canada) with a mild abrasive gel (Nuprep, Weaver and Company, Aurora, CO, USA). Conductive gel (Signa Gel, Parker Labs, Fairfield, NJ, USA) was used to lower electrode impedance below a 25Ω threshold. The participant was instructed to stare at a fixation point roughly 2 m in front of them and at eye level while the ERP stimulus was delivered using headphones.

### 2.2 ERP stimulus

An odd-ball stimulus was used to obtain the N100 and P300 responses, comprised of a standardized auditory stimulus of alternating tones and narrated word pairs used to quantify the N100, P300 and N400 responses concurrently. The oddball stimulus was consisted of a series of tones; primarily 80 dB standard tones (91% of total) with scarce 105 dB oddball tones (9% of total) [22,25]. To elicit the N400 response, word pairs (72) were semantically related, such as ‘dog’ and ‘cat’ (50%) or semantically unrelated (50%). The series of tones and word pairs alternated for a total test duration of 6 min and 15 s.

### 2.3 Data processing

Data was collected at a sampling rate of 500 Hz. The Neurocatch software (version 2.0) automatically applies 0.1–20 Hz bandpass filtering, a 60 Hz notch filter and an adaptive filter that corrects for ocular artifacts. Data was automatically normalised using detrending to account for inter-epoch drift [22,25]. Data from each trial was grand-averaged across all electrodes and displayed as a composite trace ranging from −100 ms to +900 ms from stimulus onset. ERP peaks were identified using a rater blinded to timepoint. Within participant ERP peaks were selected at once to ensure a consistent selection within-individual. The same peak was used to characterise the amplitude and latency of the response, estimated in reference to a 0 μV baseline and 0 ms (stimulus onset). N100 peaks were identified as the largest negative deviation occurring between 50 and 150 ms [22,25]. The P300 was identified as the largest positive deviation occurring between 250 and 450 ms and occurring later than the standard P200 [22,25]. When distinct P300a and P300b response were both available and had similar amplitudes, the P300a response was selected. The N400 was selected as the largest amplitude negative deviation occurring in between 350 ms and 600 ms, distinct from the PNM response and bookended by the P200 and P600 responses [22,25].

### 2.4 Assessment of exposures

At the same timepoint as each neurological evaluation, a survey was completed which assessed exposure to potential confounds. Caffeine, nicotine, alcohol, psychoactives and medication consumption in the previous 24 h was assessed via self-report questionnaire. Participants also reported changes in consumption relative to normal levels. Self-report levels of mood (very low, low, good, very good) and sleep (<4 h, 4–6 h, 6–8 h, >8 h) were also collected.

## 3 Statistical analysis

Mean and 95% confidence intervals (95% CI) were calculated for each ERP outcome, stratified by sex. Conditional linear regression models with Kenward-Roger degrees of freedom approximation were fit for each ERP outcome to assess the association between timepoint (days of riding) and the amplitude/latency of the respective ERPs, conditioned on individual. This allowed for the estimation of mean ERP outcomes for each timepoint using all available data even when a participant was missing a timepoint. Mean marginal difference between baseline observations and post-ride observations were calculated for each ERP amplitude and latency, which was estimated without contribution from those without a baseline timepoint observation. Alpha was set *a priori* at 0.05, and all analysis was performed using Stata 19 (StataCorp LLC, USA).

## 4 Results

Nineteen participants completed multiple days of ERP assessments at baseline and/or following a day of racing. Four participants sustained a suspected concussion between baseline and follow up timepoints and were excluded from the analysis. Thus, a total of 15 participants (9 male, 6 female) with a mean age of 19 years (range 15–24 years) contributed 38 observations to the analysis (see Table 1). Observation timepoints are described in table, stratified by sex. Sample timepoint characteristics are listed in Table 2. No participants reported sleeping less than 4 h, and less than 17% of the sample reported sleeping less than 6 h in all study timepoints. Caffeine and alcohol consumption was reported as the same as usual in 70% or more of the observations during each timepoint. Nicotine consumption was reported as the same as usual in all participants at every timepoint. In depth exploration of baseline ERP outcomes in MTB riders is available elsewhere [25]. Mean ERP characteristics from baseline observations are displayed in Table 3. Table 4 demonstrates the mean marginal comparison of post ride observations with baseline observations for all ERP outcomes. Table 5 demonstrates the different in N400 amplitude from baseline to following each day of riding.

**Table 1:** Distribution of the observations between sexes from baseline to days since baseline.

Time point	Female (n)	Male (n)
Baseline observations	3	8
Day 1	5	9
Day 2	3	3
Day 3	0	3
Day 4	1	3
Total	12	26

**Table 2:** Timepoint Sample Characteristics. Levels of daily factors, and whether those factors were less, the same and more than usual.

		Days of riding				
		Baseline	1	2	3	4
		n (%)				
Sleep	<4 h	1 (9.1)	0 (0)	0 (0)	0 (0)	0 (0)
	4–6 h	1 (9.1)	2 (14.3)	1 (16.7)	0 (0)	0 (0)
	6–8 h	7 (63.6)	9 (64.3)	3 (50.0)	2 (66.7)	3 (75.0)
	<8 h	2 (18.2)	3 (21.4)	2 (33.3)	1 (33.3)	1 (25.0)
	Less	3 (27.3)	3 (21.4)	1 (16.7)	0 (0)	0 (0)
	Same	8 (72.7)	10 (71.4)	5 (83.3)	1 (33.3)	4 (100.0)
	More	0 (0)	1 (7.1)	0 (0)	2 (66.7)	0 (0)
Caffeine	None	4 (36.4)	11 (78.6)	3 (50.0)	1 (33.3)	3 (75.0)
	1-2 beverages	7 (63.6)	3 (21.4)	3 (50.0)	2 (66.7)	1 (25.0)
	Less	1 (9.1)	3 (21.4)	0 (0)	0 (0)	0 (0)
	Same	8 (72.7)	10 (71.4)	6 (100.0)	3 (100.0)	3 (75.0)
	More	2 (18.2)	1 (7.1)	0 (0)	0 (0)	1 (25.0)
Mood	Very low	0 (0)	0 (0)	0 (0)	0 (0)	1 (25.0)
	Low	1 (9.1)	0 (0)	0 (0)	0 (0)	0 (0)
	Good	9 (81.8)	8 (57.1)	6 (100.0)	2 (66.7)	3 (75.0)
	Very good	1 (9.1)	6 (42.9)	0 (0)	1 (33.3)	0 (0)
	Less	0 (0)	0 (0)	1 (16.7)	0 (0)	0 (0)
	Same	10 (90.9)	10 (71.2)	5 (83.3)	2 (66.7)	4 (100.0)
	More	1 (9.1)	4 (28.6)	0 (0)	1 (33.3)	0 (0)
Alcohol	None	7 (63.6)	13 (92.9)	6 (100.0)	3 (100.0)	4 (100.0)
	1-2 drinks	2 (18.2)	1 (7.14)	0 (0)	0 (0)	0 (0)
	3-4 drinks	1 (9.1)	0 (0)	0 (0)	0 (0)	0 (0)
	5+ drinks	1 (9.1)	0 (0)	0 (0)	0 (0)	0 (0)
	Less	1 (9.1)	0 (0)	0 (0)	0 (0)	0 (0)
	Same	8 (72.7)	13 (92.9)	6 (100.0)	3 (100.0)	4 (100.0)
	More	2 (18.2)	1 (7.1)	0 (0)	0 (0)	0 (0)
Nicotine	No	9 (81.8)	14 (100.0)	6 (100.0)	2 (66.7)	3 (75.0)
	Yes	2 (18.2)	0 (0)	0 (0)	1 (33.3)	1 (25.0)
	Less	0 (0)	0 (0)	0 (0)	0 (0)	0 (0)
	Same	11 (100.0)	14 (100.0)	6 (100.0)	3 (100.0)	4 (100.0)
	More	0 (0)	0 (0)	0 (0)	0 (0)	0 (0)
Psychoactives	No	10 (90.9)	14 (100.0)	5 (83.3)	2 (66.7)	3 (75.0)
	Yes	1 (9.1)	0 (0)	1 (16.7)	1 (33.3)	1 (25.0)
	Less	2 (18.2)	0 (0)	0 (0)	0 (0)	0 (0)
	Same	9 (81.8)	14 (100.0)	6 (100.0)	2 (66.7)	4 (100.0)
	More	0 (0)	0 (0)	0 (0)	1 (33.3)	0 (0)

**Table 3:** Mean baseline Event Related Potentials (ERP) amplitudes and latencies for the N100, P300 and N400 ERPs, displayed stratified by sex and combined.

Measure	Male (n = 8)	Female (n = 3)	Combined (n = 11)
	Mean (95% CI)		
N100 amplitude (mV)	4.16 (2.92, 5.40)	5.57 (4.15, 6.99)	4.55 (3.59, 5.51)
N100 latency (ms)	100.25 (85.10, 115.40)	96.67 (86.32, 107.01)	99.27 (88.95, 109.60)
P300 amplitude (mV)	6.01 (3.96, 8.05)	6.22 (2.72, 9.71)	6.06 (4.62, 7.50)
P300 latency (ms)	280.00 (243.31, 316.69)	259.33 (135.39, 383.28)	274.36 (244.78, 303.95)
N400 amplitude (mV)	3.98 (2.72, 5.24)	4.86 (0.41, 9.30)	4.22 (3.18, 5.26)
N400 latency (ms)	391.50 (333.18, 449.82)	370.67 (332.72, 408.61)	385.82 (345.80, 425.84)

**Table 4:** Mean marginal difference of Event Related Potential (ERP) amplitudes and latencies for the N100, P300 and N400 ERPs from baseline to post-ride timepoints following a downhill mountain biking competition.

Measure	Difference estimate	95% CI	p-value
N100 amplitude (μV)	0.66	−0.70, 2.03	0.340
N100 latency (ms)	−4.48	−18.42, 9.46	0.529
P300 amplitude (μV)	0.51	−1.28, 2.29	0.577
P300 latency (ms)	−7.56	−31.57, 16.44	0.537
N400 amplitude (μV)	**−1.42**	**−2.36, −0.48**	**0.003**
N400 latency (ms)	9.23	−34.67, 53.12	0.680

**Table 5:** Association between days of downhill mountain bike riding and N400 Amplitude (μV).

Day	Change (95% CI)	*P* Value
Baseline (ref)	–	–
1	**−1.40 (−2.44, −0.36)**	**0.011**
2	**−1.46 (−2.85, −0.07)**	**0.040**
3	−1.23 (−2.96, 0.50)	0.155
4	**1.58 (−3.16, −0.01)**	**0.049**

**Fig. 2:**
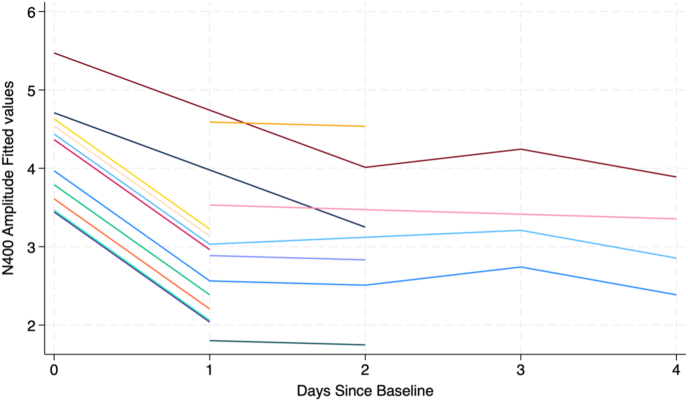
Fitted values for repeat N400 Amplitude (μV) observations after each day of downhill mountain biking. Each line represents one participant.

Mean marginal comparison between baseline and post-ride neural processing outcomes demonstrated similar observations for N100 amplitude and latency, P300 amplitude and latency and N400 latency across timepoints (all p > 0.340; Table 4). A mean marginal reduction of 1.42 μV was observed in N400 amplitudes collected post-ride compared to baseline (95%CI: −2.36, −0.48, p = 0.003; Table 4). Moreover, relative to baseline, N400 amplitudes demonstrated statistically significant reductions on days 1, 2, and 4, but not 3 of riding (Table 5, Fig. 2).

## 5 Discussion

The present study investigated the association between MTB and neural processing in youth. Results demonstrated a significant decrease in cognitive neural processing performance, evidenced by a reduced N400 amplitude post ride compared to baseline. Sensory or attentional processing (N100 and P300) was not significantly different between post-ride and baseline time points. Moreover, N400 amplitude reduction was sustained across post-ride observations. Differences in N400 amplitude are representative of changing neurocognitive resource allocation in response to a task. Interestingly, a reduced amplitude could indicate a decrease in the neural activity, requirement to interpret information, or a reduction in neural capacity to produce the response. Consequently, a recent study using blood oxygen level dependent MRI found that adults with a mTBI within the last month exhibited higher neural activation to a simple memory task compared to controls, but a smaller increase in activation with increasing task complexity [26]. This data suggests increases in neural activity in response to a similar task may indicate acute neural injury, but that a reduced response to more complex stimulus may indicate a reduced capacity for neural processing. In the present study, N100 and P300 responses, which involve lower stimulus complexity elicited similar responses in baseline compared to post injury (Table 4). However, the more integrated and demanding N400 response was reduced post-ride (Table 4). This may indicate that simple neural processing was robust to mountain biking exposure, but that the capacity for the brain to process more complex inputs is reduced, however, more evidence is required to confirm these findings.

There are multiple possible explanations for the association between MTB and the observed decrease in N400 amplitude. A study assessed HAE exposure in downhill mountain bikers using accelerometers mounted behind the right ear, and observed a mean of 30.3 HAEs greater than 10 g per run during bouts of competition riding [4]. This method of assessing HAEs is likely to overestimate exposure of the brain to HAEs due to poor coupling of the sensor to the skull [27]. Nevertheless, this evidence suggests that downhill mountain bikers may experience an accumulation of HAEs comparable to contact sports, such as rugby [28]. It has been proposed that exposure to repetitive HAEs increases neural susceptibility to SRC [8,9]. The biological mechanism underlying this theory is unclear, but it has been suggested that each HAE may result in ionic flux, temporarily impairing neural function, and potentially sensitizing the neuron to subsequent injury [29]. If the decrease in N400 amplitude observed in this study is related to the accumulation of HAEs sustained during competition mountain biking, then the N400 may represent a plausible biomarker for the acute neurophysiological sensitization to concussion caused by accumulated HAEs during sport participation. Future research is needed to confirm the biological mechanisms of sport-related changes in neurological function.

A study in youth football detected an increase in N400 latency following a season relative to baseline. This difference was associated with HAE accumulation, measured using helmet accelerometry [5]. While helmet accelerometry is also limited compared to instrumented mouthguards [27], this evidence suggests a change in neural function associated with HAEs and detectable with ERPs. Furthermore, while a study in youth ice hockey detected concussion-related impairments in the N100, P300 and N400 ERPs, they also detected an increase in N400 latency from pre-to post-season in uninjured players [19]. A recent case–control study compared adults who participate in mixed martial arts (MMA) to controls and found that those with a history of MMA participation exhibited decreased N400 amplitudes compared to controls [21]. Another case–control study that tightly matched high school students in boarding schools demonstrated that exposure to a season of participation in contact sport such as rugby and ice hockey relative to participation in non-contact sport like tennis was associated with higher N400 latency and lower N100 amplitude [22]. Taken together, it is plausible that the observed decrease in N400 amplitude in the present study is related to the HAEs accumulated during the days of downhill mountain bike racing. Future research that assesses HAE exposure and neural activation in the same participants is needed to clarify this relationship in MTB populations.

Conversely, the cognitive demand of MTB may have contributed to the observed findings. Elite downhill mountain bikers can reach speeds above 50 km/h while riding [30]. At these speeds, riders cover technical terrain quickly and must process information about the terrain and obstacles rapidly. The riders must take in visual and proprioceptive inputs at a quick rate and coordinate their riding to match the course they are racing to maximize performance and minimize the risk of injury. Furthermore, changing mid-race factors such as loss of traction requires immediate reactions to avoid crashing. This constant updating of sensory inputs throughout a ride depends on effective neural processing. Moreover, integration of sensory inputs with knowledge of the course, position and conditions is likely required to optimise performance and reduce risk of crashing. Thus, a day of riding may be neurologically demanding, leading to decreased neural processing performance as a result of fatigue rather than dysfunction. There is evidence of semantic satiation in the N400 response, whereby the repetition of the first (or prime) word of a triplet of words 30 times before a congruent and non-congruent word elicited a smaller N400 amplitude response compared to when the prime word was only repeated 3 times. While this reduction in neural response to repeated stimulus may suggest a fatiguability of the N400 response [31], assessments of the N400 following bouts of sustained focus are presently lacking. Thus, it is unclear whether sustained focus during downhill mountain bike riding influenced the observed difference in N400 amplitudes from baseline to post-ride.

Another potential influence on post-ride ERP measures observed in the current findings could be exposure to exercise itself. The baseline measures were collected following competition registration, whereas each of the post-ride measures were collected following a day of riding. Thus, it is possible the acute influence of exercise may have contributed to the differences observed in the present study. There is evidence of reduced N2 and P3 amplitudes in response to a go-no-go paradigm in physically exhausted search and rescue personnel [32]. Conversely, there is evidence that exertion testing increases the amplitude of the N400 in trained and untrained youth [33]. While the reduction of N400 amplitude observed following MTB in the present study may mirror the neural dysfunction in the exhausted search and rescue personnel, it is contrary to the expected influence of exercise on the N400. Taken together, it is unlikely that the reduction in N400 amplitude observed in the current study is explained by the influence of exercise alone.

The results of the present study may also inform the application of ERP assessment following injury in MTB athletes. MTB participation is highly injurious. A study of competitive mountain bikers reported an overall rate of 38.3 medical attention injuries per 1000 race hours [34]. The national estimate of yearly mountain bike-related visits to United States emergency departments increased from under 10,000 a year in 2014 to over 15,000 a year in 2022, with adolescents and young adults representing over 50% of all visits [35]. Moreover, head injuries represent the second most common injury site, besides the shoulder, and concussions represent over 10% of all medical attention injuries sustained during competition [34]. There is evidence that mountain biking related SRCs are most prevalent among adolescents (age 14–19 years) [35], and competitive riders are at four times the odds of sustaining an SRC [36]. Moreover, there is currently no standardized assessment protocol for suspected concussion in MTB [37]. Athlete awareness of concussion is also quite low, with as many as 68% of downhill mountain bike riders continuing to ride after experiencing SRC symptoms following a crash [36]. A meta-analysis of 278 studies reported a mean time to symptom resolution of 14 days following SRC [38] and the concussion in sport group consensus guidelines suggest a minimum 8–10 day graduate recovery process [39]. However, a study in competitive MTB reported a mean time loss from SRC of 5 days [34], suggesting that downhill mountain bikers are likely returning to sport before complete recovery. Thus, downhill mountain bikers represent a population of athletes at particular risk of SRC and its consequences, due to high rates of injury, scant medical support, lack of official concussion protocols, and low levels of athlete awareness around concussion. There is ongoing research attempting to address this critical need for objectivity in concussion assessment. Recent efforts span a variety of advanced measures such as blood biomarkers [40], transcranial ultrasonography [41], magnetic resonance imaging [42] and EEG [43] to assess pathophysiological markers of SRC. While ERP assessments are an emerging and promising avenue for the objective quantification of concussion [19], the potential for the neurological consequences of acute sport participation to confound the interpretation of clinical ERP outcomes may limit the widespread use of ERPs as a point-of-care diagnostic tool. The present study did not detect changes in N100 and P300 amplitude and latencies, or N400 latencies post-ride compared to baseline, potentially suggesting that these ERP outcomes may be robust to downhill mountain bike participation. Thus, concussion-related changes in these outcomes may be clinically interpreted without confounding by the potential influences of exercise, fatigue and accumulated HAE exposure sustained during non-concussive sport participation. Future research is required to characterise the differences in the acute response of ERPs following SRC, relative to regular sport participation, to improve the clinical utility of this outcome measure as an objective biomarker of concussion-related neurological impairment in downhill mountain bikers.

The present study detected neural processing differences following days of competition mountain biking. Studies which concurrently assess ERPs and exposure to HAEs using gold standard measures such as instrumented mouthguards are needed to assess the relationship between HAE accumulation and neural processing in this population. Expansion of evidence on the acute influence of exercise and sustained focus on ERPs is also needed to better inform the overall influence of sport participation on neural processing. Lastly, research collecting point-of-care assessments of ERPs following SRC must consider the potential for non-concussive sport exposure to influence ERP outcomes.

## 6 Limitations

The present study has some limitations. While the post-ride observations provided an assessment of the acute effects of MTB on neurophysiological outcomes, the sub-acute and long-term influences of MTB participation on neurophysiological outcomes were not evaluated. Multiple neurological exposures occurred unmeasured that may have influenced changes in N400 amplitude, including HAEs [5], exercise [33] and concentration when riding during competition.

The ERP assessment technique in the present study was limited by the number and location of electrodes, which limits spatial resolution of the ERP responses. However, this approach allowed for point of care assessments that would not have been feasible using more spatially resolute EEG. Moreover, ERP amplitudes were determined via grand-average response of repeated trials. Grand averaging of ERP waveforms prior to selecting peaks limits interpretation, as decreases in amplitude can represent consistently lower amplitude across trials, or desynchrony of responses of the same amplitude. Thus, the interpretation of an ERP amplitude decrease cannot in principle distinguish an inconsistent yet unchanged magnitude of response from a stable yet decreased magnitude response [44].

The reduced sample size limited the investigation of potentially relevant covariates such as sex, age, and stimulant consumption [25]. However, the strength of the within-participants design was that differences in individual factors did not influence the comparison of baseline and post-ride ERPs. Furthermore, not all athletes completed a baseline assessment. Thus, mean marginal differences between baseline and post-ride timepoint for the N400 ERP component could only be calculated from those who completed a baseline and post-ride timepoint, further reducing the effective sample size of this comparison.

## 7 Conclusion

The current study observed cognitive neural processing impairments following as little as one day of competitive MTB indexed by the N400 ERP. N400 amplitude was lower following days of MTB relative to baseline, but was stable across number of competition days completed. Further work is required to investigate the confounding influence of exercise and concentration when riding on changes in ERP latency and amplitudes. The N400 ERP may be a promising outcome to assess neurological function following sport participation.

## Declaration of competing interest

The authors declare that they have no known competing financial interests or personal relationships that could have appeared to influence the work reported in this paper.

## References

[1] Bailey R. (2006 Oct). Physical education and sport in schools: a review of benefits and outcomes. J Sch Health.

[2] Emery C., Tyreman H. (2009 Sep 1). Sport participation, sport injury, risk factors and sport safety practices in Calgary and area junior high schools. Paediatr Child Health.

[3] Statistics Canada (2018). https://www150.statcan.gc.ca/.

[4] Hurst H.T., Atkins S., Dickinson B.D. (2018 Dec 1). The magnitude of translational and rotational head accelerations experienced by riders during downhill mountain biking. J Sci Med Sport.

[5] Fickling S.D., Poel D.N., Dorman J.C., D'Arcy R.C.N., Munce T.A. (2022 Apr 1). Subconcussive changes in youth football players: objective evidence using brain vital signs and instrumented accelerometers. Brain Commun.

[6] Bernick C., Shan G., Ritter A., Ashton N.J., Blennow K., Lantero-Rodriguez J. (2023 Oct 12). Blood biomarkers and neurodegeneration in individuals exposed to repetitive head impacts. Alzheimers Res Ther.

[7] Kmush B.L., Mackowski M., Ehrlich J., Walia B., Owora A., Sanders S. (2020 May 1). Association of professional football cumulative head impact index scores with all-cause mortality among national football league players. JAMA Netw Open.

[8] Beckwith J.G., Greenwald R.M., Chu J.J., Crisco J.J., Rowson S., Duma S.M. (2013 Apr). Head impact exposure sustained by football players on days of diagnosed concussion. Med Sci Sports Exerc.

[9] Broglio S.P., Lapointe A., O'Connor K.L., McCrea M. (2017 Oct 1). Head impact density: a model to explain the elusive concussion threshold. J Neurotrauma.

[10] Smirl J.D., Peacock D., Wright A.D., Bouliane K.J., Dierijck J., Burma J.S. (2020). An acute bout of soccer heading subtly alters neurovascular coupling metrics. Front Neurol [Internet].

[11] Wallace C., Smirl J.D., Zetterberg H., Blennow K., Bryk K., Burma J. (2018 Aug 27). Heading in soccer increases serum neurofilament light protein and SCAT3 symptom metrics. BMJ Open Sport Exerc Med.

[12] Merchant-Borna K., Asselin P., Narayan D., Abar B., Jones C.M.C., Bazarian J.J. (2016 Dec). Novel method of weighting cumulative helmet impacts improves correlation with brain white matter changes after one football season of sub-concussive head blows. Ann Biomed Eng.

[13] Kuzminski S.J., Clark M.D., Fraser M.A., Haswell C.C., Morey R.A., Liu C. (2018 Feb 1). White matter changes related to subconcussive impact frequency during a single season of high school football. Am J Neuroradiol.

[14] Hurst H.T., Hancock S., Hardwicke J., Anderson E. (2020 Dec 31). Does participation in downhill mountain biking affect measures of executive function?. J Sci Cycl.

[15] Davis P.A. (1939 Nov). Effects of acoustic stimuli on the waking human brain. J Neurophysiol.

[16] Sutton S., Tueting P., Zubin J., John E.R. (1967 Mar 17). Information delivery and the sensory evoked potential. Science.

[17] Kutas M., Federmeier K.D. (2011). Thirty years and counting: finding meaning in the N400 component of the event related brain potential (ERP). Annu Rev Psychol.

[18] Kutas M., Hillyard S.A. (1980 Jan 11). Reading senseless sentences: brain potentials reflect semantic incongruity. Science.

[19] Fickling S.D., Smith A.M., Pawlowski G., Ghosh Hajra S., Liu C.C., Farrell K. (2019 Feb 1). Brain vital signs detect concussion-related neurophysiological impairments in ice hockey. Brain.

[20] Broglio S.P., Moore R.D., Hillman C.H. (2011 Oct). A history of sport-related concussion on event-related brain potential correlates of cognition. Int J Psychophysiol Off J Int Organ Psychophysiol.

[21] Munce T.A., Fickling S.D., Nijjer S.R., Poel D.N., D'Arcy R.C.N. (2024 Sep 12). Mixed martial arts athletes demonstrate different brain vital sign profiles compared to matched controls at baseline. Front Neurol.

[22] D'Arcy R.C.N., McCarthy D., Harrison D., Levenberg Z., Wan J., Hepburn A. (2024 Nov 22). An objective neurophysiological study of subconcussion in female and male high school student athletes. Sci Rep.

[23] Shill I.J., West S.W., Sick S., Schneider K.J., Wiley J.P., Hagel B.E. (2023 Nov). Differences in injury and concussion rates in a cohort of Canadian female and male youth Rugby Union: a step towards targeted prevention strategies. Br J Sports Med.

[24] Harris P.A., Taylor R., Minor B.L., Elliott V., Fernandez M., O'Neal L. (2019 Jul 1). The REDCap consortium: building an international community of software platform partners. J Biomed Inform.

[25] Neill M.G., Fletcher E.K.S., Larson E., Fraser K., Ramsay S., Smirl J.D. (2025 Jan). Neurophysiology of Downhill Mountain bike Athletes—Benchmark assessments of event-related potentials. Sensors.

[26] McAllister T.W., Sparling M.B., Flashman L.A., Guerin S.J., Mamourian A.C., Saykin A.J. (2001 Nov 1). Differential working memory load effects after mild traumatic brain injury. Neuroimage.

[27] Wu L.C., Nangia V., Bui K., Hammoor B., Kurt M., Hernandez F. (2016 Apr). In vivo evaluation of wearable head impact sensors. Ann Biomed Eng.

[28] Tooby J., Owen C., Sawczuk T., Roe G., Till K., Phillips G. (2025 Jun 6). Instrumented mouthguards in men's Rugby league: quantifying the incidence and probability of head Acceleration Events at a Group and individual level. Sports Med.

[29] Giza C.C., Hovda D.A. (2014 Oct). The new neurometabolic Cascade of Concussion. Neurosurgery.

[30] Hurst H.T., Swarén M., Hébert-Losier K., Ericsson F., Atkins S., Homlberg H.C. (2013). GPS-Based evaluation of activity profiles in elite downhill Mountain biking and the influence of course type. J Sci Cycl.

[31] Ströberg K., Andersen L.M., Wiens S. (2017 Dec 5). Electrocortical N400 effects of semantic satiation. Front Psychol.

[32] Zhao S., Ait-Belaid K., Shen Y., Zhou K. (2024 Oct 1). The neurological effects of acute physical exhaustion on inhibitory function. Physiol Behav.

[33] Magnié M.N., Bermon S., Martin F., Madany-Lounis M., Suisse G., Muhammad W. (2000 May). P300, N400, aerobic fitness, and maximal aerobic exercise. Psychophysiology.

[34] Palmer D., Florida-James G., Ball C. (2021 Oct). Enduro world series (EWS) Mountain biking injuries: a 2-year prospective study of 2010 riders. Int J Sports Med.

[35] Koehne N.H., Locke A.R., Yendluri A., Parsons B.O., Waterman B.R., Alaia M.J. (2025 Jan 30). Sex- and age-specific analysis of Mountain biking injuries: a 10-Year review of national injury data. Orthop J Sports Med.

[36] Clark G., Johnson N.A., Saluja S.S., Correa J.A., Delaney J.S. (2021 Nov). Do Mountain bikers know when they have had a concussion and, do they know to stop riding?. Clin J Sport Med.

[37] McLarnon M., Boyce S.H., Fisher N., Heron N. (2022 Jan). ‘It's all downhill from here’: a scoping review of sports-related concussion (SRC) protocols in downhill Mountain biking (DHI), with recommendations for SRC policy in professional DMB. Int J Environ Res Publ Health.

[38] Putukian M., Purcell L., Schneider K.J., Black A.M., Burma J.S., Chandran A. (2023 Jun). Clinical recovery from concussion-return to school and sport: a systematic review and meta-analysis. Br J Sports Med.

[39] Patricios J.S., Schneider K.J., Dvorak J., Ahmed O.H., Blauwet C., Cantu R.C. (2023 Jun). Consensus statement on concussion in sport: the 6th international conference on concussion in sport–amsterdam, October 2022. Br J Sports Med.

[40] Tabor J.B., Penner L.C., Galarneau J.M., Josafatow N., Cooper J., Ghodsi M. (2024 Sep 5). Plasma biomarkers of traumatic brain injury in adolescents with sport-related concussion. JAMA Netw Open.

[41] Neill M.G., Burma J.S., Miutz L.N., Kennedy C.M., Penner L.C., Newel K.T. (2024 Apr 8). Transcranial doppler ultrasound and concussion-supplemental symptoms with physiology: a systematic review. J Neurotrauma.

[42] Tabor J.B., Brett B.L., Nelson L., Meier T., Penner L.C., Mayer A.R. (2023 Jun 1). Role of biomarkers and emerging technologies in defining and assessing neurobiological recovery after sport-related concussion: a systematic review. Br J Sports Med.

[43] Burma J.S., Lapointe A.P., Wilson M., Penner L.C., Kennedy C.M., Newel K.T. (2023 Nov 4). Adolescent sport-related concussion and the associated neurophysiological changes: a systematic review. Pediatr Neurol.

[44] Dames K.D., Smith J.D., Heise G.D. (2017 Jun 26).

